# Predicting the Drug Safety for Traditional Chinese Medicine through a Comparative Analysis of Withdrawn Drugs Using Pharmacological Network

**DOI:** 10.1155/2013/256782

**Published:** 2013-04-29

**Authors:** Mengzhu Xue, Shoude Zhang, Chaoqian Cai, Xiaojuan Yu, Lei Shan, Xiaofeng Liu, Weidong Zhang, Honglin Li

**Affiliations:** ^1^Shanghai Key Laboratory of New Drug Design, State Key Laboratory of Bioreactor Engineering, School of Pharmacy, East China University of Science and Technology, Shanghai 200237, China; ^2^Department of Phytochemistry, School of Pharmacy, Second Military Medical University, Shanghai 200433, China

## Abstract

As the major issue to limit the use of drugs, drug safety leads to the attrition or failure in clinical trials of drugs. Therefore, it would be more efficient to minimize therapeutic risks if it could be predicted before large-scale clinical trials. Here, we integrated a network topology analysis with cheminformatics measurements on drug information from the DrugBank database to detect the discrepancies between approved drugs and withdrawn drugs and give drug safety indications. Thus, 47 approved drugs were unfolded with higher similarity measurements to withdrawn ones by the same target and confirmed to be already withdrawn or discontinued in certain countries or regions in subsequent investigations. Accordingly, with the 2D chemical fingerprint similarity calculation as a medium, the method was applied to predict pharmacovigilance for natural products from an in-house traditional Chinese medicine (TCM) database. Among them, Silibinin was highlighted for the high similarity to the withdrawn drug Plicamycin although it was regarded as a promising drug candidate with a lower toxicity in existing reports. In summary, the network approach integrated with cheminformatics could provide drug safety indications effectively, especially for compounds with unknown targets or mechanisms like natural products. It would be helpful for drug safety surveillance in all phases of drug development.

## 1. Introduction

Drug safety is always a major problem during all the phases of drug development, and its importance has been emphasized in recent years since some approved drugs have to be withdrawn due to severe adverse effects even in the postmarketing phase [1–7]. Although the Food and Drug Administration (FDA) would do drug safety surveillances by report collections on FDA drug safety communications and make consequent decisions on such approved drugs with unexpected safety problems including warnings and withdrawals [8], it should be more efficient for patients and pharmaceutical industry to minimize therapeutic risks if predictive approaches could be used to assess drug safety in the preclinical phase. In fact, there are already some drug safety predictive approaches developed to this end, which can be roughly divided into quantitative methods and qualitative methods. For the former, toxicologically based QSARs are a typical method to estimate the toxicity of new compounds using the model of a training set of chemicals with known drug-target interactions [9, 10]. Besides, knowledge-based toxicogenomics is also seen as a potent technology, which describes the toxicity of a compound through analyzing responses of the whole genome to the compound at the protein, DNA, or metabolite level and can combine measurements of cheminformatics, bioinformatics, and systems biology [11]. However, there is an obvious limitation to reduce the uses of these methods in *in silico* toxicological predictions; that is, they greatly depend on abundant high-quality experimental data [12]. Thus qualitative methods, especially network approaches are beginning to thrive in this region [13].

A network is defined as a bipartite graph consisting of nodes to represent molecular objectives and edges to deduce interactions between nodes, which can describe complex interaction events like polypharmacology in a thorough way [14, 15]. Thus, from the network-based viewpoint, toxicity prediction can be described as the identification of novel unexpected drug-target interactions by network topology analysis, machine learning algorithms, cheminformatics, and bioinformatics measurements [16–18]. Till now, there have already been a few methods developed based on network approaches. For example, Campillos et al. constructed a side-effect similarity network to identify common protein targets of unrelated drugs, which is only applicable for marketed drugs with detailed side-effect information [19]. Moreover, Cami et al. developed a predictive pharmacosafety networks (PPNs) which trains a logistic regression model to predict unknown adverse drug events from existing contextual drug safety information [20]. In addition, Yamanishi et al. investigated the relationship between chemical space, pharmacological space, and topology of drug-target interaction networks to develop a new statistical method to predict unknown drug-target interactions, which could be extended to obtain pharmacological information for test datasets with drug candidates based on their chemical structures [21, 22]. Although such existing network approaches are not perfect, it seems quite promising that they are appropriate for drug safety studies and even could be used routinely at all phases of drug discovery. There is hence a great incentive to develop improved network-based methods capable of detecting drug side effects efficiently.

Despite of these predictive methods mentioned above, there have not been special concerns on safety surveillance against medicinal natural products. As we know, traditional Chinese medicine (TCM) has been used in multiple clinical therapies for over 3,000 years, but even till now, there are still sparse research data on effective compositions, biological mechanisms, and adverse drug reactions derived by TCM formulas. Although TCM is regarded as an enormous source for drug discovery which contributes to a lot of anti-inflammatory drugs and anticancer ones, it does not mean that TCM is absolutely safe [23–28]. Presently, therapeutic risks by TCM components have been reviewed owing to the notorious aristolochic acids which were originally used to treat arthritis, rheumatism, hepatitis, and diueresis for a long time but were lately discovered to cause irreversible nephropathy and cancer in humans [29, 30]. Thus it reminds us that the critical problem of pharmacovigilance on active ingredients from TCM formulas should be focused immediately.

To this end, in this study, we firstly constructed a series of networks based on data from the DrugBank database and applied network topology analysis integrated with cheminformatics for drug safety indications [31], including drug-target networks composed by approved drugs with targets and withdrawn drugs with targets, respectively, and a drug-drug network consisting of approved drugs and withdrawn drugs by the same targets. Then 47 approved drugs with potential therapeutic risks were identified and 34 of them were verified to have already been withdrawn in some countries and regions or in the discontinued phase by reports collection. Ultimately, the approach was applied for an in-home traditional Chinese medicine (TCM) database to indicate pharmacovigilance on natural products extracted from TCM [32]. As a result, 75 natural products with potential risks in therapy were uncovered, especially a drug candidate for hepatitis C virus infection therapy in phase III, Silibinin. Therefore, our method integrating network topology analysis with cheminformatics was proved to be powerful and could provide useful pharmacovigilance indications. Particularly, it is viable to investigate TCM-derived drug safety problem.

## 2. Materials and Methods

### 2.1. Datasets

 The file for target records, the SDF files of chemical structures annotated with other properties for approved drugs, withdrawn drugs, and small molecular drugs were obtained from the DrugBank database, whereas the structural data for natural products were obtained from the in-home TCM database. Then the small molecular drugs data were used to filter the approved drugs data and the withdrawn drugs data to remove nonsmall molecular drugs. SMILES and 2D chemical structural fingerprint data for the approved drugs, the withdrawn drugs, and the natural products were prepared by a cheminformatics platform ChemAxon's suite (Academic Version) with default parameters, respectively. Local python scripts were written to format the data files appropriate for network constructions. 

### 2.2. Network Constructions and Network Topology Analysis

 drug-target networks consisting of the approved drugs with targets and the withdrawn drugs with targets were constructed with Cytoscape (Version 2.8.2), respectively [33, 34]; then approved drugs and withdrawn drugs by the same targets were identified and integrated into a new network. In particular, for the withdrawn drugs, existing reports were investigated to find out decision factors on drug withdrawals and exhibited by way of a network. Then for the approved drug-target network and the withdrawn-target network, general topology properties were analyzed by Cytoscape, respectively, while CentiBiN (Version 1.4.3) was applied to calculate common vertex centralities for each drug node in the two networks separately [35]. Furthermore, degree distribution profile of drug nodes in each network was analyzed by statistics. 

### 2.3. Cheminformatics Application

 In the withdrawn drugs-approved drugs network, such withdrawn drugs that contacted with more than 8 approved drugs were selected for cheminformatics studies, where pairwise fingerprint similarity values would be calculated by Tanimoto coefficient measurements [36, 37]. With the definition by Formula (1), *a* and *b* represented occurrences of “1” in binary coded strings *i* and *j*, respectively, while *c* was the occurrence of “1” in the same bit of the two ones. Using 0.7 as the cutoff, the approved drugs greatly similar to certain withdrawn drugs would be picked out for further investigations. Analogously, fingerprint similarity values of natural products from the TCM database against all approved drugs and withdrawn drugs from the DrugBank database were calculated, respectively. For each natural product, hit withdrawn drugs or approved ones would be recorded only when the corresponding Tanimoto coefficients were more than 0.7:

(1) $$\begin{array}{c} \text{Si}{\text{m}}_{\text{Tanimoto}}=\frac{c}{a+b-c}. \end{array}$$

## 3. Results 

### 3.1. General Network Topology Analysis on Drug-Target Networks

**Figure 1:**
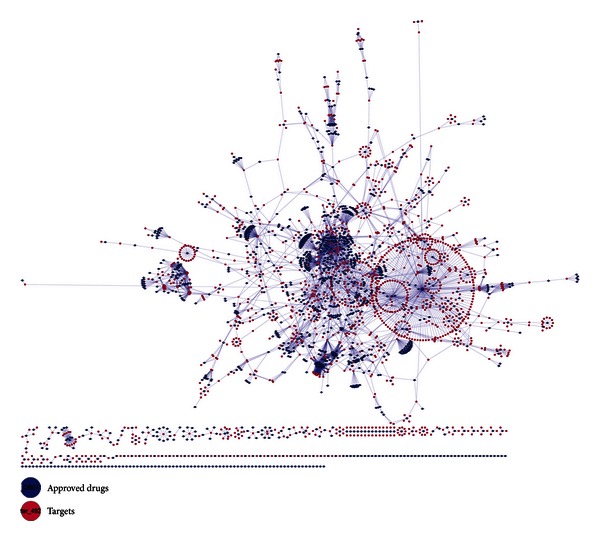
The network of approved drugs to targets from DrugBank, in which approved drugs are shown with blue filled spots and targets are exhibited by magenta ones.

**Figure 2:**
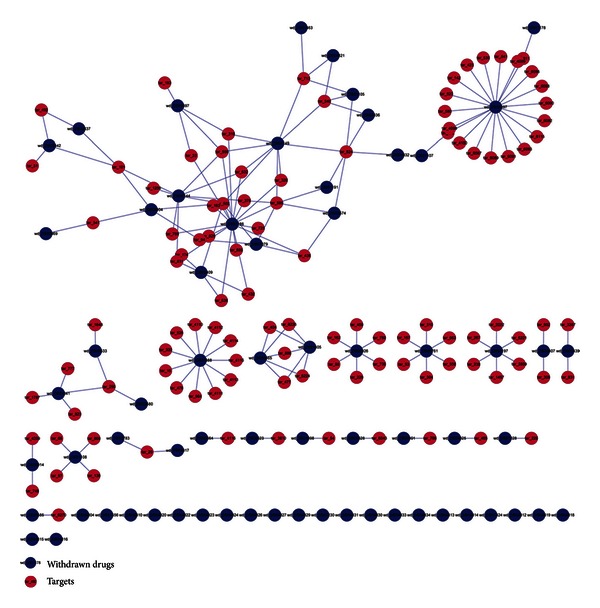
The network of withdrawn drugs to targets from DrugBank, where withdrawn drugs are represented by blue filled spots and targets are shown with magenta ones.

According to existing information from the DrugBank database, we constructed the approved drug-target network and the withdrawn drug-target network, respectively (Figures 1 and 2). For the former, there are 2889 nodes in total including 1411 drug nodes, whilst there are totally 172 nodes involving 66 drug nodes for the latter. Then general topology properties of the two networks were analyzed for comparisons (Table 1), which adopted multiple characteristics to describe the profile of a network based on graph theory like connectivity, heterogeneity, and centrality [38, 39]. In particular, some topology properties like the shortest paths value obviously depended on the size of each network, so only the ones which are unrelated to sizes or can be regularized by ratios would be discussed below. 

In details, isolated nodes in the two networks were noticed first, which made the graphs not connected. Furthermore, it was observed that they were all drug nodes without any associated target nodes. Twenty-three isolated nodes (34.85% of 66 drug nodes) were found in the withdrawn drug-target network, whereas the approved drug-target network possessed a fewer portion of 9.28% (131 ones out of 1411 drug nodes). Besides, all 172 nodes in the withdrawn drug-target network constituted 44 connected components, which on the other hand meant that a connected component had nearly 4 nodes on average, while 2889 ones in the approved drug-target network only made up 217 ones (averagely 13 nodes in a connected component). This observation virtually reflected that nodes in the approved drug-target network were prone to possess more contacts to each other. Moreover, the withdrawn drug-target network had smaller measurements of the characteristic path length (2.914) and the network diameter (7) but a larger network centralization value (0.107), whilst the ones for the approved drug-target network were 7.510, 23, and 0.054. Theses results together implicated that the withdrawn drugs were more centralized to fewer targets, even no targets, while approved drugs were more discrete to more targets. Consistent with the surmise above, the higher network density (0.011), the lower network heterogeneity (1.345) and average number of neighbors (1.849) for the withdrawn drug-target network also suggested that nodes in this network are inclined to be crowded in certain regions with fewer interactions to each other and thus exhibited lower irregularity. 

### 3.2. Vertex Centralities Studies on Drug-Target Networks

**Figure 3:**
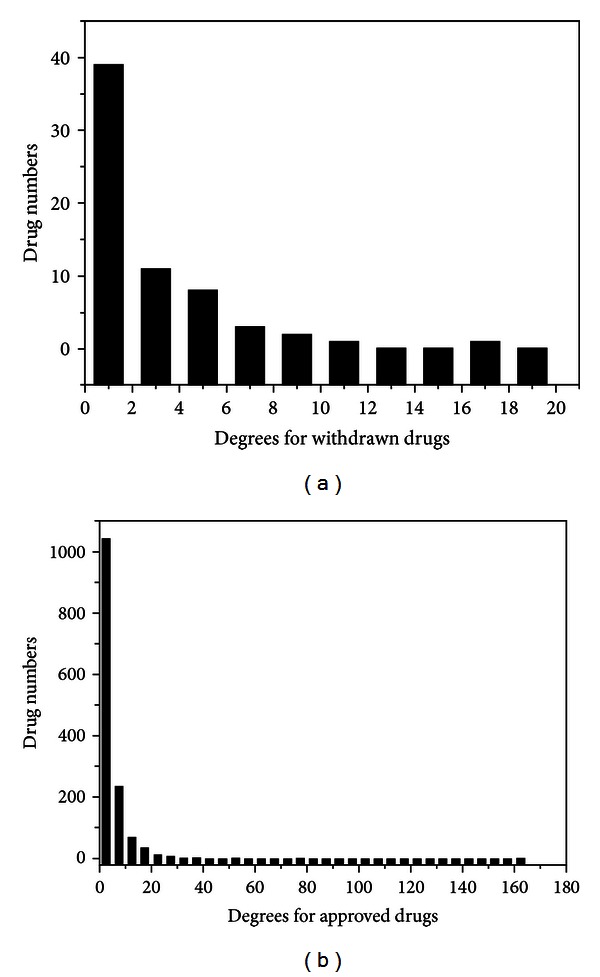
Degree distribution statistics for nodes of withdrawn drugs (a) and approved drugs (b) in corresponding drug-target networks, respectively.

Furthermore, common vertex centralities for nodes in the two networks were calculated by CentiBin to find more clues [40, 41]. Firstly, degree for each node was measured, and the ones for drug nodes were extracted for degree-distribution investigations in statistics. as illustrated in Table 2 and Figure 3, the withdrawn drug nodes had lower degrees on average and most of them (59.09%) only contacted to 2 target nodes, even less, which validated the lower network heterogeneity and average number of neighbors for the withdrawn drug-target network measured aforementioned from another point of view. By contrast, approved drug nodes had more interactions with targets, where 63.58% of them contacted to more than 2 targets, especially 1.84% of them could form associations to more than 20 target nodes.

To calculate other vertex centralities planted in the CentiBiN, the two networks were formatted into connected graphs according to the indications by CentiBiN; thus, isolated nodes and unconnected components were removed and only 45 nodes (17 drug nodes) and 2382 ones (1120 drug nodes) were retained, respectively. Consequently, data from the two networks decreased greatly, especially the one from the withdrawn drug-target network, which was insufficient for further studies but still implied an inferior connectivity of the withdrawn drug-target network. The detailed vertex centralities and network topology properties with corresponding definitions would be listed in Table S1 in the Supplementary Material available online at http://dx.doi.org/10.1155/2013/256782.

Moreover, further surveys were taken to detect corresponding diseases and therapeutic intentions derived by certain target nodes in the two connected graphs. Firstly, 28 common ones were extracted, which were almost neurotransmitter receptors or transporters distributing in central and peripheral nervous systems and regulated by agonists or antagonists to treat diseases including depression, Parkinson's syndrome, hypertension, and arrhythmia. Although degrees of these target nodes differed greatly, they acted as “bottlenecks” in the two networks and the connectivity of the graphs would be destroyed if without them. In particular, target nodes with degrees both high in the two networks implied that these targets had attracted plenty of research attentions, but risks of results in failures were high as well, whilst the ones with both lower degrees represented a deficiency of corresponding drugs. Then target nodes which only existed in the connected graph for the approved drug-target network were extracted for similar investigations. Among them, highly connected target nodes (“hubs”) represented such therapeutic intentions to develop antibacterial, anti-inflammatory, and antineoplastic agents, which also implied a lower risk of failure for involved drugs.

### 3.3. 2D Fingerprint Similarity Measurements in the Derived Drug-Drug Network

**Figure 4:**
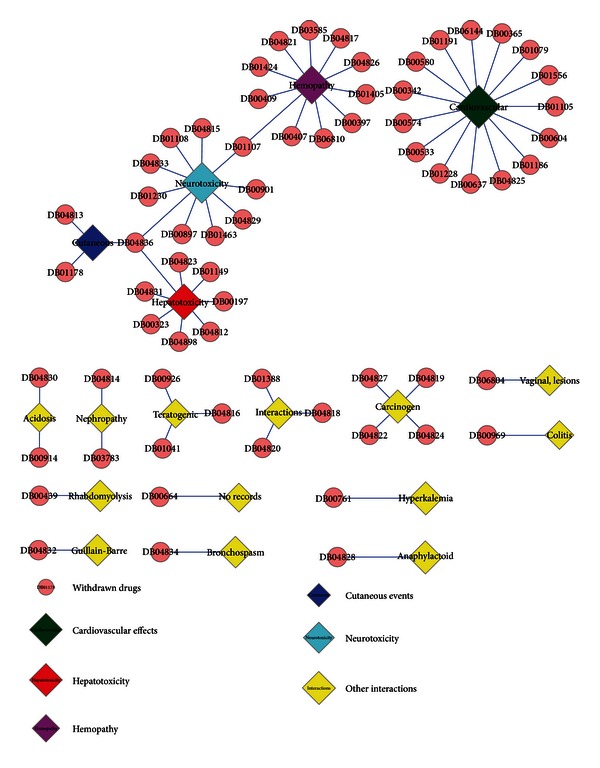
The network of critical factors resulting in drug withdrawals to the 66 withdrawn drugs from DrugBank, in which withdrawn drugs are shown with pink filled spots while adverse-effect records for them are exhibited by filled diamonds with different colors.

First of all, decisions on the 66 withdrawn drugs by FDA were collected to find out reasons for withdrawals. Although detailed withdrawal dates for them differed greatly ranging from 1961 to 2010, it was found that all of them were withdrawn due to severe unexpected adverse effects in postmarketing surveillances (Table S2). As a result, the withdrawn drug adverse effect network was built. As illustrated in Figure 4, it was clear that adverse events like hemopathy, cardiovascular toxicity, neurotoxicity, and hepatotoxicity occurred most frequently, whilst each drug mainly induced one adverse event, only two drugs resulted in syndromes of multiple side effects.

**Figure 5:**
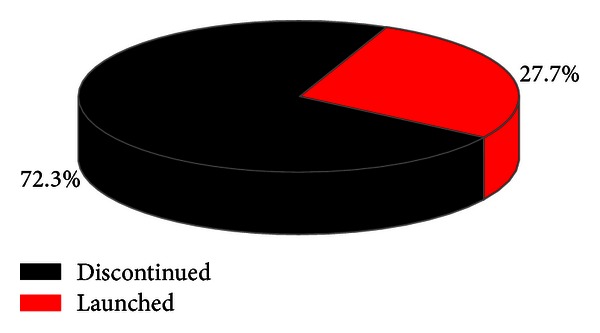
Proportion statistics of 47 approved drugs discontinued versus still launched which are greatly similar to withdrawn ones with similarity values more than 0.7, in which discontinued ones are shown in black with launched ones in red.

To explore potential toxicities derived by approved drugs for pharmacovigilance studies, 2D chemical fingerprint similarity calculations were introduced into the derived network for assistance, which involved withdrawn drugs associated with approved drugs by the same targets. Since the sum of both drugs against the same target somewhat reflected the research interests, withdrawn drugs would be selected for similarity measurements only when they contacted with more than 8 approved drugs. With the Tanimoto coefficient of 0.7 as the cutoff, 47 approved drugs were found greatly similar to withdrawn ones by the same targets in chemical property topologies. Among them, 34 ones (72.3%) have been reported to be in the discontinued phase or even banned in some countries and regions such as Europe, USA, and Canada, while 13 ones (27.7%) are still available in the market but with explicit warnings and strict indications in uses issued by FDA (Figure 5). The detailed remarks on them would be exhibited in Table S3 in the supplementary material.

Besides, side effects occurring frequently for each approved drug of the 47 ones with potential risks were collected from a side effect database SIDER, which contains information on marketed medicines and their recorded adverse drug reactions extracted from dispersed public documents [42]. Consistent to our investigations above, most of the drugs were found to have more than one adverse event records with frequencies in a descending order, especially the ones which had been discontinued. These results together reaffirmed the severe drug safety problem in the postmarketing phase, and it should be an important subject in the process of drug development. The side-effect collections with highest frequencies for the 47 approved drugs are given in Table S3 in the Supplementary Material as well.

### 3.4. Drug Safety Indications on Natural Products

The in-house traditional Chinese medicine (TCM) database contains a collection of 2156 natural products isolated from 85 medicinal plants which contributed to several DPP-IV inhibitors by target fishing and RSK2 inhibitors by a virtual screening (unpublished) [32]. Most natural products in the database have physiological activity records but with unknown targets to elucidate mechanisms of action. Thus, we mapped these natural products onto the approved drug-target network and the withdrawn drug-target network by 2D chemical fingerprint similarity calculations to all drugs, respectively. With the same definition and cutoff aforementioned, despite of 988 natural products which did not show high-similarity measurements to approved drugs or to withdrawn drugs, 1092 natural products (50.65%) were found only similar to the approved drugs greatly, which suggested that they might exhibit lower toxicities. Contrarily, 1 natural product was found only alike to the withdrawn drugs. Particularly, 75 ones (3.48%) possessed highly similar features both to the approved drugs and the withdrawn drugs, which highlighted pharmacological activities and potential toxicities simultaneously. Consequently, the 75 natural products were extracted from the database for further investigations, which would be given in Table S4 with records for similar withdrawn drugs in the Supplementary Material.

We searched PubMed and ScienceDirect for published reports about the 75 natural products, but only 25 ones had corresponding literature. Among them, Silibinin which is currently under phase III clinical trials for hepatitis C virus infection therapy had drawn more attentions. In the 2D fingerprint similarity measurements above, Silibinin was found to possess similar chemical features not only to the following approved drugs: Nabilone which is used for control of nausea and vomiting caused by chemotherapeutic agents in the treatment of cancer [43–45], Hesperetin, that is, a cholesterol-lowering flavanoid with antioxidant, anti-inflammatory and anticarcinogenic activities [46–48], and Propafenone which is used in the treatment of atrial and ventricular arrhythmias [49, 50], but also to the withdrawn drug Plicamycin, that is, an antineoplastic antibiotic but has been withdrawn for a dose-related bleeding syndrome [51–53].

In accordance with the similarity calculation results, Silibinin is a flavonolignan extracted from milk thistle seeds, which showed antiviral efficacy, anti-inflammatory activity, anticancer effects, protection against experimental ischemic stroke, and inhibition of A*β* peptide aggregation with unclear mechanisms [54–60]. Presently, there are no reports on signs of severe toxicity induced by Silibinin, but transient hyperbilirubinemia and mild sensation of heat with infusion were found as the most relevant drug-associated side effects [61, 62]. Combined with our study, although Silibinin was regarded as a promising drug candidate with a lower toxicity in existing researches, it should be highlighted with the adverse event on hemopathy in progressive clinical surveillances, especially for the old and infants or people with weak metabolism abilities.

## 4. Discussion

Modern drugs were originally designed for specific targets according to the traditional “one drug, one target, one disease” paradigm. However, drugs may exhibit “off-target” pharmacology, which would result in new therapeutic intentions or unexpected side effects. Thus, network pharmacology model was advocated to elucidate the complexity of biological interaction processes by way of drug-target networks, drug-disease networks, and so on [15]. In this study, based on discrepancies between the approved drug-target network and the withdrawn drug-target network reflected by multiple network topology properties, it is obviously observed that approved drugs tend to contact with more targets, each of which can interact with 3.891 targets on average. In the meantime, “hot” targets only connected by approved drugs mainly distribute in areas of antibacterial, anti-inflammatory, and antineoplastic therapies, while the ones both associated with approved drugs and withdrawn drugs locate in central and peripheral nervous systems for the treatment of diseases such as depression, Parkinson's syndrome, hypertension, and arrhythmia. This observation would give useful guidance for the research intentions on concrete targets considering risks of failure for involved drugs. Accordingly, combining the results together, it is clear that the network based approach could provide useful information to retain a balance between multiple targeted drug design and adverse side effects, especially in certain therapeutic areas. 

Besides, integrated with 2D chemical fingerprint similarity calculations, the two drug-target network models could be used to predict potential targets or pharmacovigilance for marketed drugs or unknown compounds based on their chemical structures. In particular, we investigated drug safety problems induced by natural products extracted from known medicinal plants. As we know, natural products have played a pivotal role in the development of chemotherapies, but for most ones, both bioactive mechanisms and safety boundaries are not known very clearly. Thus with structural chemical features as mediums, mapping of natural products onto the two drug-target networks would provide more information for elucidations. In cases of natural products from the in-house TCM database, it is observed that they exhibit certain diversity in structures (45.83% of them with Tanimoto coefficient <0.7 against all drugs) which would largely expand the chemical space, whilst 54.17% of them could be selected by chemical similarity measurements for potential pharmacovigilance explorations. 

From a technical viewpoint, for the similarity calculation, if 3D geometric shape measurements or other sophisticated functions such as the ones using information mining of medical literature could be considered [63–66], the performance of the method would be better or it would provide all-around information. However, in fact, active conformations of most drugs interacting with targets are not ascertained well. Thus, it could be another major problem to calculate similarity values between conformation sets which would not only exhaust computational sources but also introduce more uncertainties. Moreover, there are few available pharmacology reports on natural products which make it difficult to adopt text mining approaches. Therefore in our studies herein, we tried to simplify these problems and just indicated potential therapeutic risks by drugs qualitatively. 

Lastly, compared to adverse drug reaction prediction methods based on sole similarity measurements such as the famous similarity ensemble approach (SEA) [67–71], our methods combined both the advantages of network analysis and cheminformatics, which can not only discover potential side-effect events but also reveal topology properties of targets corresponding to disease modules or pathways. Therefore, it would provide all-around information for drug safety surveillance from the viewpoint of network pharmacology instead of a sole target.

## 5. Conclusion

To detect the discrepancies between approved drugs and withdrawn drugs, drug-target information from the DrugBank database was regularized to construct drug-target networks, and network topology measurements were taken to analyze them. In particular, connectivity-corresponding properties like degrees, connected component numbers, and average neighbor numbers were highlighted because they differentiated each other greatly, which suggested that approved drugs were prone to interact with more targets on average. Then, the withdrawal reasons of these withdrawn drugs were investigated, and it was found that all of them failed due to unexpected adverse effects. Besides, with the chemical structures of withdrawn drugs as probes, 2D fingerprint similarity calculations were adopted for approved drugs and natural products from an in-house TCM database for pharmacovigilance studies, respectively. Consistent with sequent text mining from existing reports, this method was found efficient to provide drug safety indications, especially for compounds with unknown targets or mechanisms like natural products. It is the first time that pharmacovigilance on natural products in large scale is focused and evaluated by network-based approaches. We believe that the network approach integrated with cheminformatics measurement would be quite useful for drug safety surveillance in all phases of drug development.

## Supplementary Material

Table S1: Key network topology property functions planted in Cytoscape and CentiBin.
Table S2: Summary for the 66 withdrawn drugs from DrugBank.
Table S3: Decisions and side effect records on approved drugs with similarity measurements more than 0.7 to withdrawn ones by same targets.
Table S4: Natural Products from the in-house TCM database with similarity values more than 0.7 to withdrawn drugs.

## Figures and Tables

**Table 1:** The general network topology analysis on the withdrawn drug-target network and the approved drug-target network by Cytoscape.

Topology properties	Withdrawn drug-target network	Approved drug-target network
Clustering coefficient	0.0	0.0
Connected components	44	217
Network diameter	7	23
Network radius	1	1
Network centralization	0.107	0.054
Shortest paths	2902	5673774
Characteristic path length	2.914	7.510
Avg. number of neighbors	1.849	3.801
Number of nodes	172	2889
Number of drug nodes	66	1411
Network density	0.011	0.001
Network heterogeneity	1.345	1.874
Isolated nodes	23 (34.85%)	131 (9.28%)
Number of self-loops	0	0
Multiedge node pairs	0	0

**Table 2:** Degree distribution statistics of drug nodes in the withdrawn drug-target network and the approved drug-target network, respectively.

Degree distribution	Withdrawn drug nodes	Approved drug nodes
Drug nodes in total	66	1411
Average degrees	2.409 ± 3.742	3.891 ± 6.569
Degree ≤ 2	59.09%	37.42%
2 < degree ≤ 4	16.67%	30.26%
4 < degree ≤ 6	12.12%	11.91%
6 < degree ≤ 20	12.12%	18.57%
Degree > 20	0	1.84%
