# Supplementary material for: Predicting the Drug Safety for Traditional Chinese Medicine through a Comparative Analysis of Withdrawn Drugs Using Pharmacological Network
Source: Evid Based Complement Alternat Med. 2013 Apr 29;2013:256782. doi: 10.1155/2013/256782 (PMC3657406; doi:10.1155/2013/256782)
Supplement: Supplementary file 1 — Table S1: Key network topology property functions planted in Cytoscape and CentiBin. Table S2: Summary for the 66 withdrawn drugs from DrugBank. Table S3: Decisions and side effect records on approved drugs with similarity measurements more than 0.7 to withdrawn ones by same targets. Table S4: Natural Products from the in-house TCM database with similarity values more than 0.7 to withdrawn drugs. [file 256782.f1.pdf]

Supplemental material

Table S1. Key network topology property functions planted in Cytoscape and CentiBin.

Feature	Function	Description
Degree	k_i	The number of links to node i
Clustering coefficient	$2n_i / [k_i(k_i - 1)]$	n_i is the number of connected pairs between all neighbors of node i
Neighborhood connectivity	c_i	The average number of second neighbors of node i
Radiality	$\sum_{j \in V} (\Delta G + 1 - dis(i,j)) / n-1$	△G is the maximal distance amongst all distance calculated between each couple of nodes, dis(i,j) denotes the length of the shortest path between node i and node j
Topological coefficient	$avg(J(i,j)) / k_i$	J(i,j) denotes the number of sharing nodes between node i and node j
Eccentricity	$1 / \max\{dis(i,j) : j \in V\}$	The longest shortest path of node i.
Stress	$\sum_{s \neq i \in V} \sum_{t \neq i \in V} \sigma_{st}(i)$	$\sigma_{st}(i)$ denotes the number of shortest paths from s to t that i lies on.
Betweenness centrality	$\sum_{s \neq i \in V} \sum_{t \neq i \in V} \sigma_{st}(i) / \sigma_{st}$	σ_{st} denotes the number of shortest paths from s to t
Closeness centrality	$1 / avg(dis(i,j))$	dis(i,j) denotes the length of the shortest path between node i and node j
Average shortest path length	d_i	The shortest path of node i

Table S2. Summary for the 66 withdrawn drugs from DrugBank.

Accession Number	Therapy areas	Withdrawal reasons	Withdrawal date
**DB00197**	antihyperglycemic, treatment of Type II diabetes mellitus	Hepatotoxicity	2000
**DB00323**	potent, selective, and reversible inhibitor of catechol-O-methyltransferase, treatment of Parkinson's disease	Hepatotoxicity	1998 in UK.
**DB00342**	H1-receptor antagonist antihistamine,	cardiac arrhythmia	in the 1990s in

	the treatment of allergic rhinitis, hay fever, and allergic skin disorders	caused by QT interval prolongation	the USA
**DB00365**	antibacterial, treatment of adults with mild to moderate infections	cardiac events and sudden death, due to its side effect of lengthening the QT interval on the electrocardiogram	1999 in USA
**DB00397**	a sympathomimetic agent, the treatment of nasal congestion, control of urinary incontinence, priapism and obesity	hemorrhagic stroke	May 31, 2001 in Canada
**DB00407**	anticoagulant, prevention of deep vein thrombosis	excessive bleeding and bruising	March 2000 in the USA
**DB00409**	antipsychotic, the treatment of schizophrenia	incidence of aplastic anemia	1993 in UK
**DB00439**	Anticholesteremic, treatment of hypercholesterolemia and mixed dyslipidemia	Rhabdomyolysis,	August 8, 2001 in Canada
**DB00533**	anti-inflammatory, the treatment of osteoarthritis, rheumatoid arthritis, acute pain in adults, and primary dysmenorrhea, as well as acute treatment of migraine attacks with or without auras	increased risk of heart attack and stroke associated with long-term, high-dosage use	September 30, 2004 in USA
**DB00574**	the management of exogenous obesity, Anorexigenic agent	heart valve disease and pulmonary hypertension; cardiac fibrosis	1997 in USA
**DB00580**	anti-inflammatory, the treatment of osteoarthritis and dysmenorrhoea	possible increased risk of heart attack and stroke	in 2005 in Canana, USA and EUro
**DB00604**	treatment of adult patients with nocturnal heartburn due to gastroesophageal reflux disease	arrhythmias, long QT syndrome	28 July, 2000 in UK
**DB00637**	the treatment of allergy symptoms	cause arrhythmias at high doses, especially when taken with CYP inhibitors or grapefruit juice	1999
**DB00664**	Anti-Infective, used for respiratory and urinary tract infections and also for malaria	Agranulocytosis and anaemia	March 2000
**DB00761**	the treatment of hypokalemia	hyperkalemia, Late	

		manifestations include muscle paralysis and cardiovascular collapse from cardiac arrest	
**DB00897**	Anti-anxiety, the short-term treatment of insomnia	risk of psychiatric adverse drug reactions.	withdrawn in UK
**DB00901**	the treatment of asthma and COPD	problems with dependence and abuse	in 2001 in USA
**DB00914**	the reatment of type II diabetes mellitus	high incidence of lactic acidosis	in 1977 in USA
**DB00926**	treat severe psoriasis; now used to treat T-cell lymphomas	the high risk of birth defects	in 1998 in USA
**DB00969**	the treatment of symptoms of severe diarrhea-predominant irritable bowel syndrome (IBS) in women with chronic symptoms	adverse effects including ischemic colitis, severely obstructed or ruptured bowel, and death	in November 2000 in USA
**DB01041**	the acute treatment of the cutaneous manifestations of moderate to severe erythema nodosum leprosum (ENL)	teratogenic effects	in 1961 in USA
**DB01079**	treatment of women with irritable bowel syndrome (IBS)	risks of heart attack or stroke	On March 30, 2007 in USA
**DB01105**	the treatment of obesity	the risk of heart attack and stroke in patients with a history of heart disease	In October 2010 in USA and Canada
**DB01107**	treating insomnia,	Side effects can include: Skin rash, fever, depression, ulcers or sores in mouth or throat, unusual bleeding or bruising, confusion, fast heartbeat, respiratory depression, swelling of feet or lower legs, dizziness, drowsiness, headache, double vision, clumsiness, constipation, diarrhea, nausea, vomiting, unusual weakness	in June 1965 in USA and in September 1990 in Canada

**DB01108**	the treatment of Cushing's syndrome	darkening of skin, drowsiness or tiredness, loss of appetite, mental depression, skin rash, and/or vomiting	in April 1994 in USA
**DB01149**	the treatment of depression	the small possibility of hepatic (liver) injury	On May 20, 2004 in USA
**DB01178**	Used in the management of anxiety and in the treatment of muscle spasm.	confirmed serious and rare cutaneous reactions (toxic epidermal necrolysis)	in 1996
**DB01186**	the treatment of Parkinson's Disease	the risk of cardiac valvulopathy	in 2007 in USA and Canada
**DB01191**	Antiobesity, the management of obesity	cardiovascular side-effects	in 1997 in USA
**DB01228**	management of irregular heartbeats	frequent proarrhythmic side effects	on December 16, 1991 in USA
**DB01230**	treatment of Attention Deficit Hyperactivity Disorder	Side effects include insomnia, anorexia, stomach ache, skin rashes, increased irritability, mild depression, nausea, dizziness, headache, drowsiness, and hallucinations	In March 2005 in USA
**DB01388**	the treatment of angina and high blood pressure	potentially harmful interactions with other drugs	in 1998
**DB01405**	Anti-Bacterial, the treatment of lower respiratory tract infections, genital and urinary infections like prostatitis, and skin infections	temafloxacin syndrome-- hemolysis	in USA in 1992
**DB01424**	anti-inflammatory, and antipyretic properties	risk of agranulocytosis	in 1999
**DB01463**	the treatment of depressive fatigue	due to problems with dependence and abuse	
**DB01556**	Used as an appetite suppressant	pulmonary hypertension and cardiac fibrosis	
**DB03585**		bone marrow suppression	in March, 1985 in Canada

**DB03783**	analgesic, NSAID and fever reducer	nephropathy (damage to or disease of the kidney)	in June 1973 in Canada
**DB04812**	Dermatologic Agent	cholestatic jaundic	on August 5, 1982
**DB04813**	Anti-Infective Agent	serious skin disorders	on October 24, 1967
**DB04814**		nephropathy	in 1963, in Canada, USA and UK
**DB04815**	used as a topical antifungal treatment, a broad-spectrum antibacterial with antifungal properties	neurotoxicity	in 1983
**DB04816**	Cathartics	genotoxicity	Withdrawn from the Canadian, US, and UK markets in 1998
**DB04817**	used a powerful painkiller and fever reducer; NSAID	potentially fatal agranulocytosis	on June 27, 1977
**DB04818**	the treatment of depression (originally intended to treat tuberculosis)	interactions with food products containing tyrosine	in July 1964 in Canada
**DB04819**	a antihistamine and anticholinergic drug	carcinogen	in May and June 1979
**DB04820**	MAOI (monoamine oxidase inhibitor) antidepressants	interactions with food products containing tyrosine	in 1963 in Canada, USA and UK
**DB04821**	dopamine reuptake inhibitor	increased incidence of hemolytic anemia	on March 20, 1992 in USA and Canada, UK
**DB04822**	treat all types of cough of various etiologies	carcinogenicity	in 1976 in Canada, USA and UK
**DB04823**	laxative	jaundice	in 1970s
**DB04824**	laxative	carcinogenicity	in 1997 in Canada
**DB04825**	Vasodilator Agent, treat angina pectoris	cardiac arrhythmias	in 1988 in Canada, USA and UK
**DB04826**	an antihistamine with anticholinergic properties used as an antipruritic	neutropenia	in 1963 in Canada, USA and UK

**DB04827**	Antineoplastic Agent	carcinogenic	in 1963 in Canada, USA and UK
**DB04828**	the management of mild to severe pain.	fatal and near-fatal anaphylactoid reactions	in March 1983 in Canada, USA and UK
**DB04829**	an agent in psychedelic therapy,	withdrawn after it became widely used recreationally	in 1950s-1960s
**DB04830**	anti-diabetic drug	lactic acidosis	in 1978
**DB04831**	treatment of hypertension	hepatitis	in 1982
**DB04832**	the treatment of depression	risk of Guillain-Barré syndrome	in 1983
**DB04833**	sedative-hypnotic drug, the treatment of insomnia, and as a sedative and muscle relaxant	Withdrawn because of risk of addiction and overdose	in 1984
**DB04834**	Used in anaesthesia, to aid and enable endotracheal intubation	fatal bronchospasm	in 2001 in many countries
**DB04836**	the treatment of depression	hepatotoxicity, dermatological side effects, and abuse potential.	in 1999
**DB04898**	anticoagulant, the treatment of acute deep vein thrombosis	hepatotoxicity (liver damage)	in 2006
**DB06144**	antipsychotic, the treatment of schizophrenia	numerous cardiac adverse effects	in 1998
**DB06804**	used as a vaginal spermicide, contraception	a higher incidence of vaginal lesions, increase the risk of infection with sexually transmitted human papillomaviruses (HPVs) that can cause cervical cancer	
**DB06810**	the treatment of testicular cancer, Paget's disease of bone, and, rarely, the management of hypercalcemia	a dose-related bleeding syndrom	in 2000

Table S3. Decisions and side effect records on approved drugs with similarity measurements more than 0.7 to withdrawn ones by same targets.

**Withdrawn Drugs[a]**	**Similar approved drugs by the same target[b]**	**Side effect records[c]**
wd_DB00197, hepatotoxicity	app_DB00412, withdrawn in Euro, 2010, 9 and in New	Rosiglitazone, respiratory tract infection, 7.3%-9.9%

	Zealand in 2011, 4 for heart disease risks, but in USA is still allowed	
	app_DB01132, withdrawn in some countries in 2011,7, for an increased risk of bladder tumors	Pioglitazone, disease progression, 13.4%
wd_DB00342, cardiac arrhythmia	app_DB00376,	Trihexyphenidyl, no records
	app_DB00387,	Procyclidine, no records
	app_DB00505, many patients discontinued it for unwanted side effects.	Tridihexethyl, no records
	app_DB00950, adverse cardiac events including cardiac arrhythmias, discontinued only in France in 2004	Fexofenadine, vomiting, 4.2%-12%
	app_DB01146,	Diphenylpyraline, no records
	app_DB01231, currently not marketed in USA or Canada	Diphenidol, no records
wd_DB00365, cardiac arrhythmia	app_DB00218, FDA has issued many warnings about the adverse effects. last resort, discontinued in USA for some therapy areas in 2004	Moxifloxacin, nervous system disorder, 4.2%
	app_DB00467, no longer available in USA	Enoxacin, no records
	app_DB00487, extensively prescribed in France and not been approved for use in USA.	Pefloxacin, no records
	app_DB00537, FDA has issued many warnings about the adverse effects, discontinued in USA for some therapy areas in 2004	Ciprofloxacin, abdominal pain
	app_DB00685, withdrawn in Euro in 1999 for hepatotoxicity, but available in USA for very restricted indications	Trovafloxacin, dizziness, 2%-11%
	app_DB00978, banned in USA in 2008	Lomefloxacin, acidosis
	app_DB01044, banned in USA in 2006, in India in 2011	Gatifloxacin, anaphylactic shock

	app_DB01059, very strict indications, last resort	Norfloxacin, arthralgia
	app_DB01137, FDA has issued many warnings about the adverse effects, discontinued in Singapore, Romania and South Africa in 2011	Levofloxacin, application site reaction, 0.6%-16.8%
	app_DB01155, withdrawn in 2009 in Euro	Gemifloxacin, anaphylactic shock
	app_DB01165, FDA has issued many warnings about the adverse effects, discontinued only in USA in 2012 for bacterial infection	Ofloxacin, application site reaction, 0.6%-16.8%
	app_DB01208, banned in USA in 2000	Sparfloxacin, no records
wd_DB00397, hemorrhagic, stroke	app_DB00368,	Norepinephrine, acidosis
	app_DB00610, discontinued by Manufacturer in 2003 in USA	Metaraminol, no records
	app_DB00852, banned or strict prescription in some countries like Australia	Pseudoephedrine, anorexia
	app_DB01364, limited used in USA and UK	Ephedrine, anorexia
	app_DB01576, discontinued by GSK, but not withdrawn	Dextroamphetamine, abdominal cramps
	app_DB06707, discontinued in USA	Levonordefrin, no records
wd_DB00574, heart valve disease	app_DB01577, scheduled drug in most countries due to its high potential for addiction and abuse, discontinued in USA	Methamphetamine, constipation
wd_DB00897, psychiatric	app_DB00404, discontinued in USA	Alprazolam, drowsiness, 41%-76.8%
	app_DB00546, discontinued in USA and Euro	Adinazolam, no records
	app_DB00683, discontinued by Roche in UK	Midazolam, vomiting, 0.75%-11%
	app_DB01215, discontinued by Abbott	Estazolam, anxiety, frequent
wd_DB00901,	app_DB00449, discontinued	Dipivefrin, arrhythmia

addiction and abuse	app_DB01408,	Bambuterol, no records
wd_DB00926,<br>birth defects	app_DB00459, discontinued for a narrow TI and a long elimination half life	Acitretin, abdominal pain
wd_DB01041,<br>teratogenic effects	app_DB00480, discontinued in Phase III	Lenalidomide, angioedema
wd_DB01107,<br>bleeding depression and vomit	app_DB00237, discontinued	Butabarbital, no records
	app_DB00312, discontinued	Pentobarbital, anaemia
	app_DB01351, discontinued in UK	Amobarbital, no records
	app_DB01353, discontinued in UK	Butethal, no records
wd_DB01186,<br>heart valve disease	app_DB00216, only by prescription in USA and Canada	Eletriptan, asthenia, 4%-10%
	app_DB00248, discontinued in Euro	Cabergoline, aggression
wd_DB01191	app_DB01577, scheduled drug in most countries due to its high potential for addiction and abuse, discontinued in USA	Methamphetamine, constipation
wd_DB01405,<br>hemolysis	app_DB00218, FDA has issued many warnings about the adverse effects. last resort, discontinued in USA for some therapy areas in 2004	Moxifloxacin, nervous system disorder, 4.2%
	app_DB00467, no longer available in USA	Enoxacin, no records
	app_DB00487, extensively prescribed in France and not been approved for use in USA. last resort	Pefloxacin, no records
	app_DB00537, FDA has issued many warnings about the adverse effects, discontinued in USA for some therapy areas in 2004	Ciprofloxacin, abdominal pain
	app_DB00685, withdrawn in Euro in 1999 for hepatotoxicity, but available in USA for very restricted indications	Trovafloxacin, dizziness, 2%-11%
	app_DB00978, banned in USA	Lomefloxacin, acidosis

	in 2008	
	app_DB01044, banned in USA in 2006, in India in 2011	Gatifloxacin, anaphylactic shock
	app_DB01059, very strict indications, last resort	Norfloxacin, arthralgia
	app_DB01137, FDA has issued many warnings about the adverse effects, discontinued in Singapore, Romania and South Africa in 2011	Levofloxacin, application site reaction, 0.6%-16.8%
	app_DB01165, FDA has issued many warnings about the adverse effects, discontinued only in USA in 2012 for bacterial infection	Ofloxacin, application site reaction, 0.6%-16.8%
	app_DB01208, banned in USA in 2000	Sparfloxacin, no records
wd_DB01463, addiction and abuse	app_DB00182, used under severe restriction, withdrawn only in Canada in 2005	Amphetamine, no records
	app_DB00191,	Phentermine, constipation
	app_DB00865, discontinued but not withdrawn	Benzphetamine, no records
	app_DB01576, discontinued by GSK, but not withdrawn	Dextroamphetamine, abdominal cramps
	app_DB01577, scheduled drug in most countries due to its high potential for addiction and abuse, discontinued in USA	Methamphetamine, constipation
wd_DB03783, nephropathy	app_DB00316, discontinued	Acetaminophen, abdominal pain
wd_DB04817, agranulocytosis	app_DB01435,	Antipyrine, no records
wd_DB04821, hemolysis	app_DB06148,	Mianserin, no records

[a]The abbreviation of “wd” represents “withdrawn”, and the drug numbers are from the Drugbank database.

[b]The abbreviation of “app” represents “approved”, and the drug numbers are from the Drugbank database.

[c]The side effect event and frequency records are from the SIDER database.

Table S4. Natural Products from the in-house TCM database with similarity values more

than 0.7 to withdrawn drugs.

**withdrawn drugs**	**targets**	**therapy int entions**	**withdrawn reasons**	**Compoun ds from TCM dat abase[a]**	**Notes**
DB04816	Cytochrome P450 1 A2	Cathartics	Withdrawn from the Canadian, US, and U K markets in 1998 d ue to geno toxicity.	NPD41	3,4-dihydroxya cetophenone
				NPD44	2,4-dihydroxya cetophenone
				NPD90	Physcion
				NPD140	2,6-Dihydroxya cetophenone
				NPD153	3,4-Dihydroxya cetophenone
				NPD330	(+)-6-hydroxy-1-methyl-2,3-di hydro-1H-Inde ne-4-carboxald ehyde
				NPD408	Sugiol
DB00926	Retinoic acid recept or alpha; Retinoic acid receptor RXR-alpha; Retinoic acid receptor beta; Reti noic acid receptor RXR-gamma; Retin oic acid receptor R XR-beta; Retinoic a cid receptor gamma -1; Cytochrome P4 50 19A1; Cytochro me P450 26A1; Ce llular retinoic acid-binding protein 1	to treat seve re psoriasis, now used t o treat T-cel l lymphoma s. It also ap pears to inh ibit NADH oxidase acti vity	It was rem oved from the Unite d States m arket in 19 98 and the Canadian market in 1996 as a psoriasis m edication, due to the high risk of birth de fects	NPD882	Difengpin-A
DB04824	UDP-glucuronosyltr ansferase 1-9	Used for ov er a century as a laxati ve	withdrawn in Canada due to con cerns with carcinoge nicity in 1 997	NPD264	ethyl-4-hydrox ybenzoate[b]
				NPD881	Ethyl p-hydrox ybenzoate[b]
DB06804	Prostaglandin G/H synthase 2	vaginal sper micide	an increase d risk of	NPD283	(-)-Secoisolaric iresinol

			opportunistic infections	NPD291	5-O-methylafzelechin
				NPD292	(-)-afzelechin
				NPD562	Secoisolariciresinol[b]
				NPD676	1-(4-Hydroxy-3-methoxyphenyl)-2-[2-methoxy-4-(3-hydroxypropyl)phenoxy]-3-propanol
				NPD685	(-)-Epiafzelechin
				NPD689	Afzelechin
				NPD876	4,4'-Dihydroxy-3,3'-dimethoxy-9,9'-epoxylignan
				NPD1150	epiafzelechin
				NPD1159	secoisolariciresinol[b]
				NPD1395	Bisphenol A diglycidyl ether
				NPD1625	4-(4-hydroxy-3-methoxyphenyl)-2-butanol
				NPD1702	3-methoxy-8,4'-oxyneoligna-3',4,9,9'-tetraol
				NPD1871	2-[4-(3-hydroxypropyl)-2- methoxyphenoxy]-propan-1,3-diol
DB06810	DNA	Treatment of testicular cancer, Paget's disease of bone, and, rarely, the management of hypercalcemia	a dose-related bleeding syndrome	NPD4	Silibinin
				NPD2197	Wistin
				NPD358	Myzodendrone
				NPD203	(2R,3S)-dihydromyricetin
				NPD207	(2R,3S)-taxifolin-3-O-arabinoside
				NPD208	(2S,3R)-taxifolin-3-O-arabinosi

					de
				NPD356	Choerospondin
				NPD351	Narirutin
				NPD295	neochamaejasmin B
				NPD294	isochamaejasmin
				NPD274	Isoneochamaejasmin A
				NPD906	11-O-vanilloyl bergenin
				NPD754	3,5,3'-Trihydroxy-7,4'-dimethoxyl-dihydroflavone
				NPD669	Dihydroquercetin
				NPD545	4-O-α-L-rhamnopyranosyl-(1→6)-β-D-glucopyranosyl acetophenone
				NPD528	4-O-β-D-glucopyranoside-acetophenone
				NPD464	Prunin
				NPD448	ormocarpin
				NPD447	Pyrroside B
				NPD445	Hemiphloin
				NPD362	Scolymoside
				NPD905	11-O-(4'-O-methylgalloyl)-bergenin
				NPD838	taxifolin
				NPD818	L-Picein
				NPD763	Davidioside
				NPD756	3,5,7,3'-tetrahydroxy-4'-methoxyl-dihydroflavone
				NPD907	11-O-(4'-hydroxybenzoyl)-bergenin

				NPD908	bergenin
				NPD1066	matairesinoside
				NPD1119	7-methoxy neo chamaejasmin A
				NPD1126	7-methoxy neo chamaejasmin B
				NPD1149	2,2'-bisteppogenin-7-O-β-glucopyranoside
				NPD1334	Osmanthuside G
				NPD1696	Dihydrosyringin[b]
				NPD1335	Dihydrosyringin[b]
				NPD1697	Pallasiin
				NPD1708	Cynanoneside A
				NPD1750	Gaultheroside A
				NPD1757	Gaultheroside G
				NPD1758	dihydrodehydrodiconiferylalcohol-9-O-b-D-oxylopyranoside
				NPD1940	1-(4-((2S,3S,4R,5S)-tetrahydro-3,4,5-trihydroxy-6-(hydroxymethyl)-2H-pyran-2-yloxy)phenyl)ethanone
				NPD2061	1-(4'-hydroxy-3'-methoxyphenyl)-2-[4"-(3-hydroxypropyl)-2"-hydroxyphenoxy]-1,3-propandiol-4'-O-β-D-xylopyranoside

				NPD1999	β-D-Glucopyranoside-4-phenylbutan-2-one
				NPD2141	(+)-lyoniresinol-3α-O-β-D-glucopyranoside
				NPD2140	(-)-lyoniresinol-3α-O-9-O-(6’-3,5-dimethoxy-4-hydroxybenzoyl)-β-D-glucopyranoside
				NPD2139	(+)-lyoniresinol-3α-O-β-D-glucopyranoside
				NPD2105	Junipetrioloside A
				NPD2074	1-(4’-hydroxy-3’-methoxyphenyl)-2-[4”-(3-hydroxypropyl)-2”-hydroxyphenoxy]-1,3-propandiol-4”c- O-α-L-rhamnoside
				NPD2071	1-(4’-hydroxy-3’-methoxyphenyl)-2-[4”-(3-hydroxypropyl)-2”-methoxyphenoxy]-1,3-propandiol-4”c-O-α-L-rhamnoside
				NPD2069	1-(4’-hydroxy-3’-methoxyphenyl)-2-[4”-(3-hydroxypropyl)-2”-hydroxyphenoxy]-1,3-propandiol-4’-O-α-L-rhamno-2”-O-β-D-glucopyranoside

				NPD2068	Styraxlignolide D
				NPD2065	1-(4’-hydroxy-3’-methoxyphenyl)-2-[2”-hydroxy-4”-(3-hydroxypropyl)phenoxy]-3-propanol-3-O-α-L-rhamno-4”c- O-β-D-xylopyranoside

[a] The “NPD” is the abbreviation of “Natural product database”, and the accession numbers are from the in-house TCM database as well.

[b] They were extracted from different herbal sources.
